# Supplementary material for: Imaging metal-like monoclinic phase stabilized by surface coordination effect in vanadium dioxide nanobeam
Source: Nat Commun. 2017 Jun 14;8:15561. doi: 10.1038/ncomms15561 (PMC5474733; doi:10.1038/ncomms15561)
Supplement: Supplementary Information — Supplementary Figures, Supplementary Notes, Supplementary Discussion and Supplementary References. [file ncomms15561-s1.pdf]

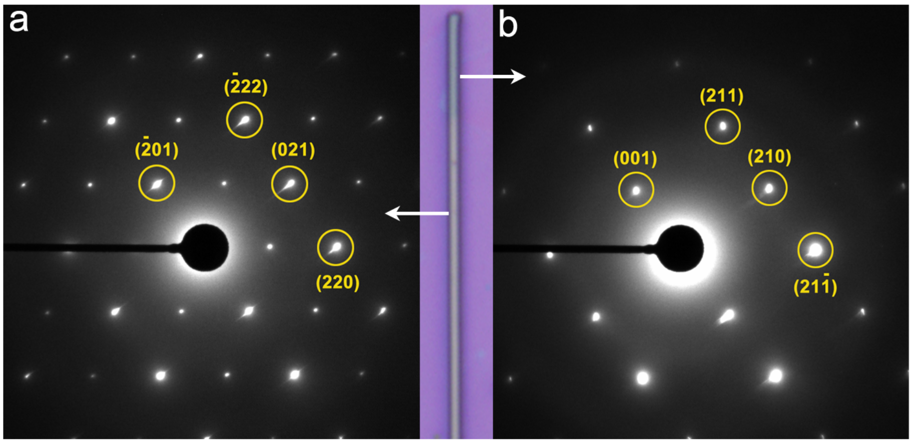

Supplementary Figure 1 | Selected area electron diffraction of a single AA-treated VO_2 nanobeam. (a), selected area electron diffraction for the bright domain. (b), selected area electron diffraction for the dark domain. The terminal dark domain can be indexed to rutile structure ($P4_2/mnm$) and the middle bright domain was assigned to monoclinic structure ($P2_1/c$).

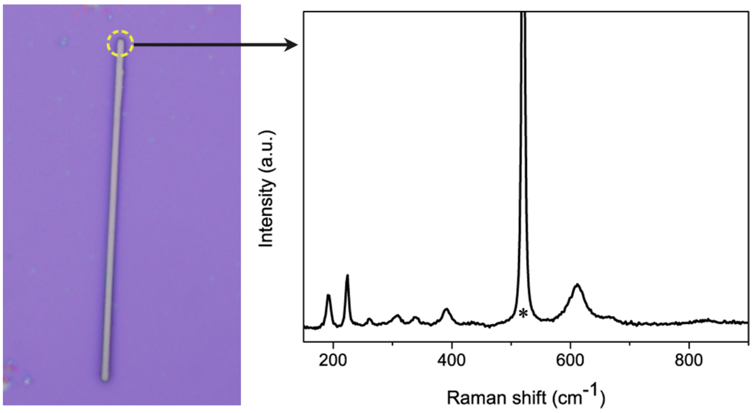

Supplementary Figure 2 | Optical image and Raman spectra of the VO_2 nanobeam treated in pure water. The peak labeled by “*” in Raman spectra belongs to the silicon substrate.

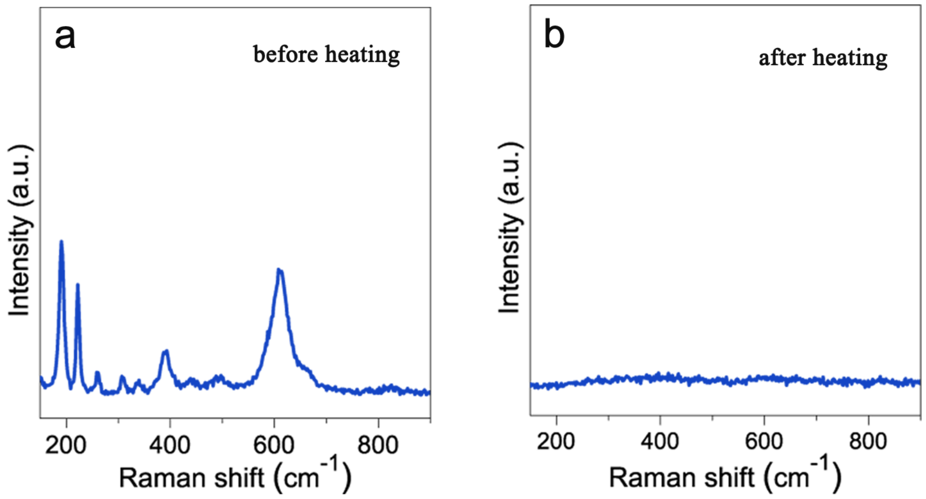

Supplementary Figure 3 | Raman spectra. (a) and (b), Room-temperature Raman spectra of this metastable region before and after the heating treatment, respectively. The measured region exhibits the characteristic peaks of VO_2 (M1) phase before heating treatment; while the Raman spectra of the same region matches well with the VO_2 (R) after heating treatment[1]. These results indicate a homogenous phase in this metastable region.

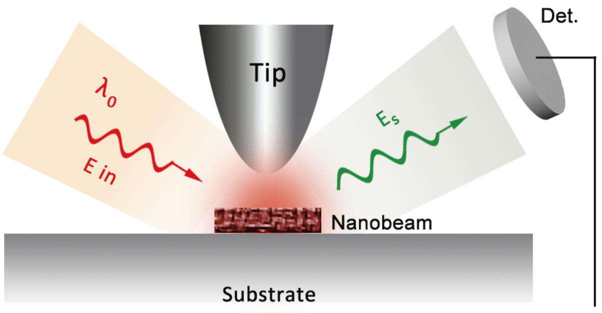

Supplementary Figure 4 | Schematic of the scanning near-field infrared microscope. The near-field images of the AA-treated VO_2 nanobeam were performed on NeaSNOM (Neaspec GmbH Co.) with the resolution of about 10 nm. The incident infrared laser beam excites metallic tip and the backscattered light is recorded to achieve the near-field infrared image of the sample.

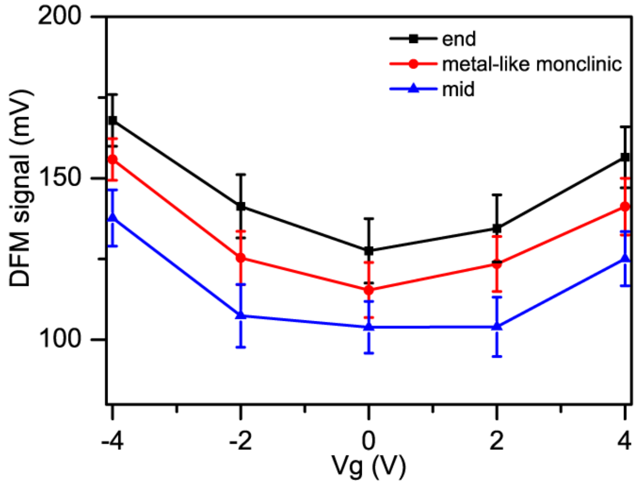

Supplementary Figure 5 | DFM signal versus V_g plot from -4V to +4V for end metallic rutile, metal-like monoclinic and mid insulating monoclinic domains. The error bars were standard deviation of DFM signal in each domains.

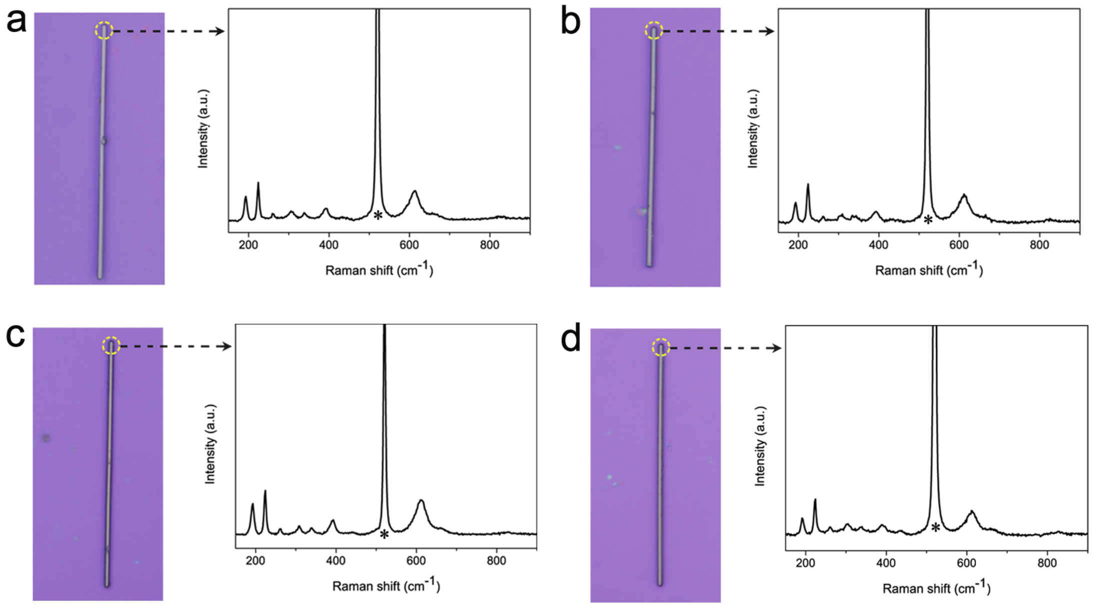

Supplementary Figure 6 | parallel experiments with other molecules. (a)-(d), Optical images and Raman spectra of the VO_2 nanobeams treated in oxalic acid, ethylene glycol, glucose and $NaBH_4$ solution. Note: the reducing ability of oxalic acid, ethylene glycol and glucose is weaker than L-ascorbic acid, while reducing ability of $NaBH_4$ is stronger than L-ascorbic acid. The peak labeled by "*" in Raman spectra belongs to the silicon substrate.

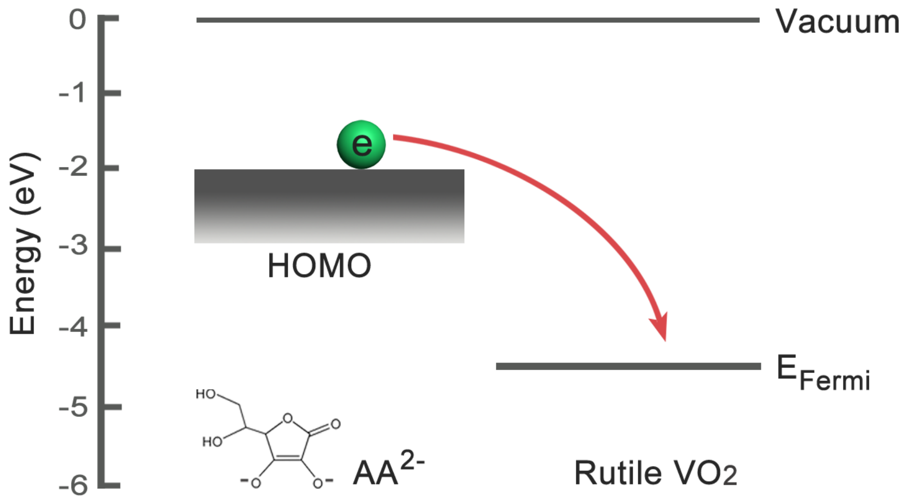

Supplementary Figure 7 | Schematic diagram shows the energy level alignment of the AA^{2-} species and rutile phase VO_2, confirming the electron transfer from the highest occupied molecular orbital (HOMO) of AA^{2-} species to rutile VO_2 nanobeam.

Supplementary Note 1

In order to confirm that the stabilization of rutile metal and metastable metal-like monoclinic phases is due to the AA treatment, the control experiments were performed. VO_2 nanobeams were treated in the pure water without adding AA and other conditions kept unchanged. As shown in **Supplementary Figure 2**, it can be seen that the VO_2 nanobeam exhibits a homogeneous bright reflection of insulating monoclinic phase and no dark domains emerged at the two ends of the nanobeam. The Raman spectra further demonstrates the VO_2 nanobeam keeps monoclinic phase at room temperature after treatment in pure water[1]. Thus, these results indicate the emergency and stabilization of rutile metal phase at room temperature is attributed to the AA molecules in our treatment.

Supplementary Note 2

As exhibited in **Supplementary Figure 5**, the quantitative DFM signal at different V_g shows that there are three different DFM response regions in all V_g, which confirms that the metal-like monoclinic intermediate phase has higher carrier density than mid phase, and lower carrier density than end phase.

Supplementary Note 3

In order to further detect how the AA molecules attached on the surface of VO_2 nanobeams, we have simulated the vibrational modes of enolic C-O connecting with the surface V atoms. After geometry optimization, the vibrational modes are calculated for free AA molecule (gas phase) and adsorbed molecule. By comparing the noticeable differences in frequencies, the structure information of the adsorbed molecule can be determined. According to the simulated vibrational modes, we found that vibration peak of the enolic C-O vibration is at 1386 cm^{-1} when connected with the V atoms. The calculated value is close to the observed new peak at 1390 cm^{-1} in our ATR-FTIR spectrum, suggesting the formation of C-O-V complexes on the surface of the AA-treated VO_2 nanobeam.

Supplementary Note 4

To inspect whether other electron-donating molecules can be utilized to trigger the phase engineering along the VO_2 nanobeams, parallel experiments of the oxalic acid, ethylene glycol, glucose, $NaBH_4$ treatment on the VO_2 nanobeams were also performed. As illustrated in **Supplementary Figure 6**, it can be clearly seen that the VO_2 nanobeams exhibit no color change and the Raman spectra indicate the VO_2 nanobeams still keep monoclinic phase after these treatments. Thus, we deem that the interaction between L-ascorbic acid and VO_2 nanobeam is a complicated process and effective regulation of VO_2 MIT using chemical molecules needs two critical factors.

First, the treated molecules could coordinately bond on the surface of VO_2 nanobeams to form a stable complex. Second, the suitable match of energy level between the chelated molecules and VO_2 is indispensable to induce the charge transfer.

Supplementary Note 5

In order to confirm that the electrons can transfer from the surface binding AA species to VO_2 nanobeam, we calculate the molecular orbital energy of the surface AA species and the Fermi level of rutile phase VO_2 as shown in **Supplementary Figure 7**. Of note, in our case, the AA treatment of VO_2 nanobeam is at the temperature of 80 °C, where the VO_2 nanobeam is in the metallic rutile phase, thus we calculate the Fermi level of the rutile VO_2. In addition, from the reaction process, we can see that the AA molecules bind to the VO_2 surface through the ortho-substituted hydroxyl groups of the enolic C-O-H and remove the two H^+ ions. In this regard, we calculate the molecular orbital energy of the surface binding AA^{2-} species (AA molecule removing two H^+ ions) as the electron transfer precursor. From the calculated results, the HOMO energy of binding AA^{2-} species is -2.0 eV and the Fermi level of rutile VO_2 is -4.5 eV compared with the vacuum level, which allows the electrons transfer from the HOMO of AA species to rutile VO_2 nanobeam.

Supplementary Discussion

About the R-phase domain grows in from the ends. From the optical and DFM images, it can be understood that the AA molecules binding on the end surface of VO_2 nanobeams contriubted electrons injection to VO_2 nanobeams. Due to the energy matching and overlap of the V3*d* orbitals along the V-V atomic chains, electrons can transfer from AA to VO_2 nanobeams along the chain direction. Thus, the R-phase domain grows in from the ends of the nanobeam with treatment time.

About the small section of metal-like monoclinic domain. In our case, the chelated AA molecules transferred electrons into VO_2 nanobeams along the V-V atomic chain direction. The electron density varies as a function of position away from the ends of AA-treated VO_2 nanobeam. The monoclinic metallic domain exists in a small part of the nanobeam between the stabilized R phase domain and initial M1 phase domain. This suggests that there may be a specifically small range of electron density necessary to stabilize metal-like monoclinic phase.

Supplementary References

1. Zhang, S., Chou, J., & Lauhon, L. Direct Correlation of Structural Domain Formation with the Metal Insulator Transition in a VO_2 Nanobeam. *Nano lett.*, **9**, 4527-4532 (2009).
